# Environmental determinants of distribution of freshwater snails and trematode infection in the Omo Gibe River Basin, southwest Ethiopia

**DOI:** 10.1186/s40249-019-0604-y

**Published:** 2019-11-20

**Authors:** Seid Tiku Mereta, Jemal Bedewi, Delenasaw Yewhalaw, Belayhun Mandefro, Yihun Abdie, Dechassa Tegegne, Wondwosen Birke, Worku Legesse Mulat, Helmut Kloos

**Affiliations:** 1 Department of Environmental Health Sciences and Technology, Jimma University, P.O. Box 378, Jimma, Ethiopia; 2 Department of Medical Laboratory Sciences & Pathology, Jimma University, P.O. Box 378, Jimma, Ethiopia; 3 Department of Biology, College of Natural and Computational Sciences, Dilla University, P.O. Box 419, Dilla, Ethiopia; 4 School of Veterinary Medicine, Jimma University, P.O. Box 307, Jimma, Ethiopia; 5 Department of Environmental Health, Wello University, P.O. Box 1145, Dessie, Ethiopia; 6 Department of Epidemiology and Biostatistics, University of California, San Francisco, USA

**Keywords:** *Biomphalaria pfeifferi*, Cercaria, Ethiopia, Freshwater, Schistosomiasis, Snail, Trematode

## Abstract

**Background:**

Determination of infection rates of snail populations is one of the basic tools for epidemiological studies of snail borne diseases. In this study, we opted to determine the trematode infection of freshwater snails in the Omo-Gibe River Basin, southwest Ethiopia.

**Methods:**

We collected snail samples from 130 observation sites in lakes, wetlands, rivers, reservoirs and irrigation canals surveyed during the dry season (March to May) in 2016. The snail samples were examined for trematode infections by cercarial shedding immediately after collection. Habitat conditions, water quality, human water contact practices and other human activities were assessed at each survey site. A redundancy analysis (RDA) was used to examine the relationship between cercarial infection and environmental variables. The statistical significance of eigenvalues and cercariae-environment correlations generated by the RDA were tested using Monte Carlo permutations at 499 permutations.

**Results:**

A total of 3107 snails belonging to five species were collected. The most abundant species was *Biomphalaria pfeifferi*, representing 66% of the total collection. Overall, 109 (3.6%) of the snails were found infected with trematodes (cercariae). *Biomphalaria pfeifferi* was found to be the most highly infected, accounting 85% of all infected snails. A total of eight morphologically different types of cercariae were recorded, which included: *Echinostoma* cercariae*,* brevifurcate apharyngeate distome cercariae, amphistome cercariae, brevifurcate apharyngeate monostome cercariae, xiphidiocercariae, longifurcate pharyngeate distome cercariae, strigea cercariae and unidentified cercariae. Brevifurcate apharyngeate distome cercariae, and *Echinostoma* cercariae were the most abundant cercariae, accounting for 36 and 34% of all infection, respectively. The mean concentration of water conductivity and 5 days biological oxygen demand were higher in irrigation canals and lake sampling points. Human activities such as open field defecation, urination, livestock grazing, farming, and swimming were highly correlated with trematode infection.

**Conclusions:**

The abundance, occurrence and infection rates of snail species were largely influenced by water physicochemical quality, sanitation and water contact behaviour of the inhabitants. Human activities, such as open field defecation and urination, livestock grazing, farming, and swimming were important predictors of the abundance of cercariae. Therefore, awareness creation should be implemented for proper containment of excreta (urine and faeces) and reducing human and animal contacts with surface waters to reduce snail-borne disease transmission.

## Multilingual abstracts

Please see Additional file 1 for translations of the abstract into the five official working languages of the United Nations.

## Background

Snail-borne parasitic diseases, such as schistosomiasis and fascioliasis, pose serious risks to human and animal health and cause major socio-economic problems in many tropical and sub-tropical countries [1]. Millions of people in approximately 90 countries have suffered from parasitic diseases in which snails serve as intermediate hosts [1]. Most of freshwater snails can serve as intermediate hosts for a number of trematode parasites [1]. Among these, the genera *Biomphalaria, Bulinus, Lymnaea*, and *Oncomelania* are important intermediate hosts for the trematode parasites *Echinostoma*, *Schistosoma* and *Fasciola* [2, 3] and play a significant role in the transmission of parasitic diseases to humans, other mammals [4] and birds [5]. In Ethiopia, the genera *Biomphalaria, Bulinus* and *Lymnea* are the medically most important intermediate host snails distributed in different parts of the country and transmit schistosomiasis and fascioliasis [6].

The larvae (cercariae) of trematodes develop in snail tissue, escape and find suitable secondary intermediate hosts or definitive hosts (human and animals) by means of passive transmission (metacercaria) or active penetration [7]. *B. pfeifferi* is the intermediate host of some trematodes. In southwest Ethiopia, *B. pfeifferi* has been reported to shed mammalian *Schistosoma* cercariae, whose presence is an indication of human intestinal schistosomiasis [8]. *Lymnaea* spp.shed amphistome cercariae which cause amphistomiasis in humans and domestic animals, mostly in cattle and sheep [9]. In addition, *Lymnaea* spp. also shed echinostome cercariae. Echinostomiasis, caused by echinostome cercariae, is an important intestinal food-borne parasitic disease in Asian countries, where humans become infected after ingesting raw or insufficiently cooked molluscs, fish, crustaceans and amphibians [10].

High prevalence and diversity of trematode infections tend to reduce snail populations. Therefore, the occurrence of some competing trematodes may be used as a biological control of snail-borne diseases [11, 12]. The prevalence and intensity of trematode infections are affected by various biological, physical and behavioural factors [13]. Habitat use and defecating habits are the main determinants of trematode infections in mud-snail populations inhabiting salt marsh ponds in Iceland [14]. In spite of the fact that the documentation of snail species and their larval trematode fauna help in our understanding of snail-borne diseases and location of potential transmission sites, studies on larval trematode infections and factors attributed to it in freshwater snails in Ethiopia are limited. Therefore, this study aims to determine the distribution of freshwater snail intermediate hosts and cercarial infection rates in Omo-Gibe River Basin, where several hydroelectric dams and irrigation canals have been constructed on the main river and tributaries which create favorable habitat for the occurrence and abundance of snail intermediate hosts.

## Methods

### Study area

**Fig. 1:**
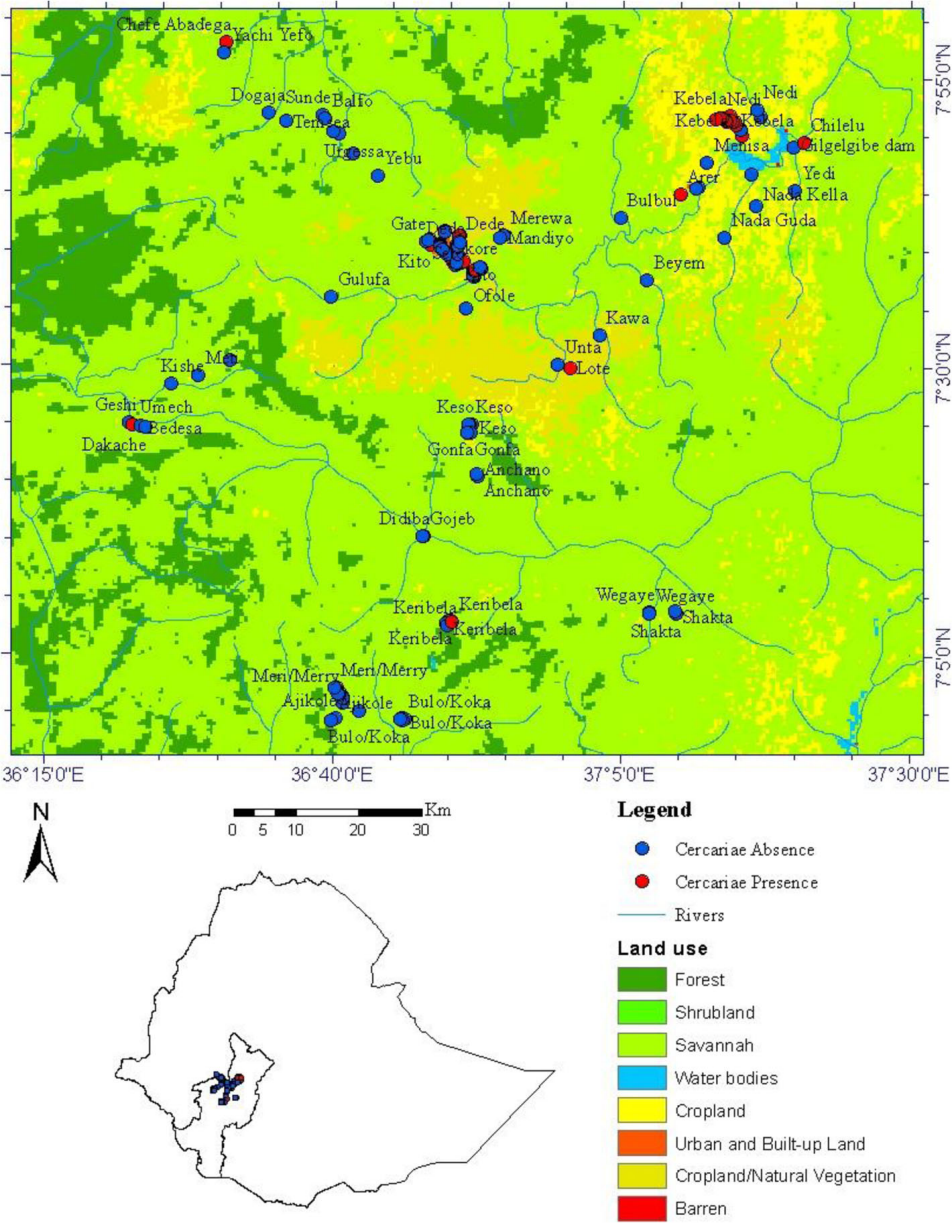
Map showing the distribution of trematode infection in the Omo-Gibe River Basin, southwest Ethiopia

This study was conducted in water bodies of Omo-Gibe River Basin situated between latitudes 4°25ˈ51.6“ N and 9°22ˈ28.05” N and longitudes 33°0ˈ24.4“ E and 38°24ˈ42.24” E. The Omo-Gibe River Basin has an area of approximately 79 561 km^2^. It is Ethiopia’s second largest river basin, accounting for 14% of Ethiopia’s annual runoff, and being second only to the Blue Nile in runoff volume [15]. Elevation data derived from Advanced Space-borne Thermal Emission and Reflection Radiometer Digital Elevation map (ASTER DEM) imagery shows that the basin has an altitude between 500 m above sea level around Lake Turkana in the south and 3000 m above sea level around Bako Town in the north. The regional climate varies from temperate wet in the highlands to hot semi-arid in the lowland, with a mean annual rainfall around 1550 mm. During recent decades, the Omo-Gibe watershed has been subjected to considerable economic and water resources development and with accelerated human population growth, hydroelectric projects and urbanization. Several dams are planned, designed and constructed on the Omo River and on the Gibe tributary. The surveyed water bodies include rivers, wetlands (Awetu, Haro, Boye), two shallow lakes (Keribela and Bulo), a hydroelectric dam (Gilgel Gibe I) and irrigation canals (Chebera Churchura) (Fig. 1).

### Malacological survey

A total of 130 sites distributed over the five types of water bodies (rivers and streams = 100 sites; wetlands = 10 sites; lakes = 10 sites; a dam = 5 sites and irrigation canals = 5 sites) were surveyed during the dry season (March to May) in 2016. Snail sampling was carried out at each site for 30 min using a scoop net with wire mesh measuring 1.5 mm on an iron frame (40 × 30 cm) and mounted on a 1.5 m long iron handle [16]. Time was allotted proportionally to cover different meso-habitats such as open water and emergent vegetation. The collected snail samples were kept in ventilated plastic buckets filled with water and vegetation from each sampling site and transported to the Laboratory of the Department of Environmental Health Sciences and Technology, Jimma University. In the laboratory, snails were maintained by feeding them fresh lettuce and spinach. Snail species were identified to species according to the morphological features of the shell described earlier [17–19].

### Examination of cercarial infection

Out of the total of 3107 snails collected, 3045 were examined for cercarial infection; the remaining 62 (2%) died during transport. Collected snails were rinsed in chlorine-free tap water to remove mud and plants. Each snail was kept in a petri-dish of 90 mm diameter containing 20 ml of dechlorinated water at room temperature (25 to 30 °C). Each petri dish was covered with perforated plastics to prevent escaping and to provide good aeration. Each snail was exposed to natural light during the day between 10:00 and 12:00 for 1 h to induce shedding of cercariae [20]. Cercariae were identified at the genus level, based on gross morphological characteristics, swimming behaviour and resting position as described by Frandsen [21] and Schell [22]. In addition, cercariae were stained with iodine solution and observed under a stereomicroscope [23]. Every day, snails were fed fresh lettuce and spinach, the water was changed, and snails were examined for emerging cercariae. Snails that did not shed cercariae in the first hour were monitored for shedding cercariae at 1 h intervals for another 24 h. Snails that did not shed cercariae were kept in glass aquaria in the laboratory and rechecked for cercariae shedding for 4 weeks. Prevalence of snail species-specific infections was determined as a percentage, by taking the number of snails that shed cercariae divided by the total number of snail species examined [24].

### Environmental variables

Multiple environmental variables were quantified at each surveyed site in each habitat type. Conductivity, pH, daytime dissolved oxygen concentration, and water temperature were measured in the field using a multi-probe meter (HQ30d Single-Input Multi-Parameter Digital Meter, Hach). Water turbidity was measured in the field using a fluorometer (Turner De-sign Aqua Fluor. A water sample (200 ml) was taken from each site and subsequently filtered through a 0.45 μm filter paper in the field to determine hardness, nitrate and orthophosphate concentrations. Unfiltered water (500 ml) was used to determine 5 day biochemical oxygen demand (BOD_5_)_._ Water samples were kept cool and in the dark during transportation to the laboratory and analysed according to the standard method [25]. In addition, the habitat type (pool or riffle) at each measurement point was subjectively assessed. The criteria for this assessment were: 1) riffle: swiftly flowing with a large proportion of its water surface broken; and 2) pool (slow flowing with a smooth water surface).

### Human activities

Human activities such as farming, grazing, open field defecation and urination, fishing, washing, irrigation, bathing and swimming were quantified based on their intensity in the studied habitats [26]. A score of 1 was given for no or minimal human activities, 2 for moderate and 3 for high human activities. Open field defecation and urination are defined here as behaviors where people defecate and urinate in places other than the toilet, such as in bushes, fields, back yards, open spaces, water bodies, and other places.

### Data analysis

A Detrended Correspondence Analysis (DCA) in CANOCO4.5 for windows (Cambridge University Press) was used to determine whether a linear (RDA) or Unimodal (CCA) type of response was present along environmental gradients [27]. The DCA yielded gradient lengths that were less than three standard deviations. In RDA analysis, cercariae infection was considered as the response variable, whereas environmental variables were treated as independent variables. All environmental data except pH were log(x + 1) transformed and standardized since the variables were measured in a variety of units. The statistical significance of eigenvalues and species-environment correlations generated by the RDA were tested using Monte Carlo permutations at 499 permutations.

Geographic coordinate readings were recorded for all sampling sites using a hand-held global positioning system unit (GPS) (Garmin GPS 60, Garmin International Inc. and Olathe, Kansas, USA). Coordinate readings were integrated into a GIS database using Arc MAP Version 10 (Environmental Systems Research Institute, New York, USA). All digital data in the GIS were displayed in the World Geodetic System (WGS) 1984 Coordinate System.

## Results

### Occurrence and abundance of freshwater snail intermediate hosts

A total of 3107 freshwater snails belonging to five species were collected from 130 sites. The species included *Biomphalaria pfeifferi* and *Biomphalaria sudanica* (intermediate hosts of *Schistosoma mansoni* causing intestinal schistosomiasis), *Bulinus globosus* (intemediate host of *S. haematobium* causing urinary schistosomiasis), *Bulinus forskalii* (intermediate host of *Schistosoma intercalatum* causing urinary schistosomiasis), *Lymnaea natalensis* and *Lymnaea truncatula* (intemediate hosts of *Fasciola gigantica* and *Fasciola hepatica* which causefascioliasis). *B. pfeifferi* was found to be the predominant snail species, accounting for 66% of the total collection and collected from 40% of the surveyed sites*. B. pfeifferi* was collected from rivers, wetlands, a lake and irrigation canals but was not encountered in the Gilgel Gibe I Reservoir. *L. natalensis* was the second most common snail species, accounting for 25% of all snails collected. It occurred in 30% of the surveyed sites and was encountered in all habitat types. *Bu. globosus* and *Bu. forskalii* accounted for less than 10% of the total collection and were mostly found in river and wetland habitats. The least common snail species was *B. sudanica,* encountered only at one river sampling site (Table 1).

**Table 1:** Relative abundance of freshwater snail fauna in the Omo-Gibe River Basin, southwest Ethiopia

Snail species	Habitat type					
	River (*n* = 100)	Wetland (*n* = 10)	Lake (*n =* 10)	Dam (*n =* 5)	Irrigation canal (*n =* 5)	Total
*Biomphalaria pfeifferi*	1968	73	2	0	29	2072
*B. sudanica*	7	0	0	0	0	7
*Lymnaea natalensis*	447	185	25	15	75	747
*Bulinus globosus*	95	23	30	0	0	148
*Bu. forskalii*	115	18	0	0	0	133
Total	2632	299	57	15	104	3107

### Cercarial infection in freshwater snails

**Plate 1:**
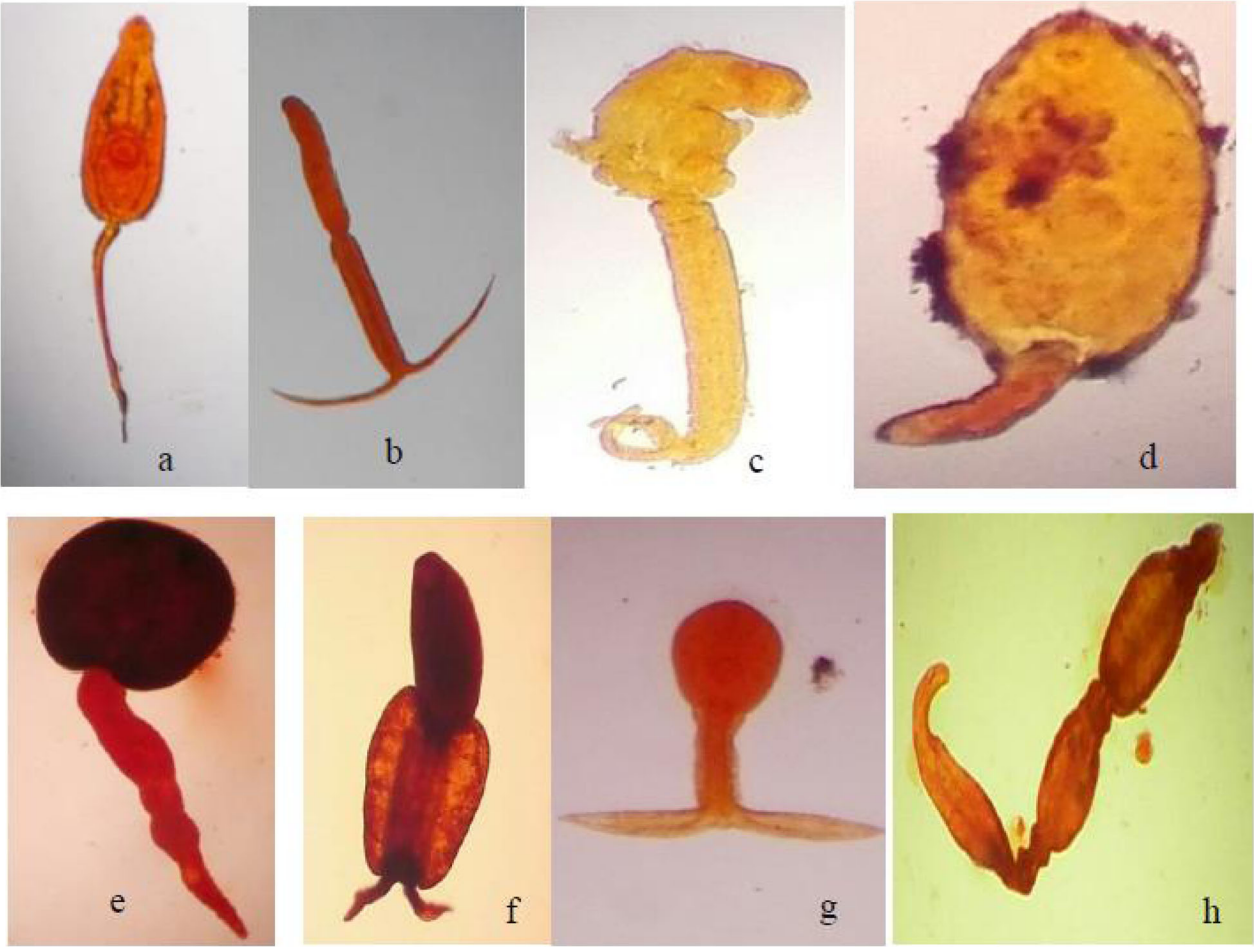
Morphotypes of trematode cercariae recorded in freshwater snails from Omo-Gibe River Basin: **a** Echinostome cercariae; **b** Brevifurcate apharyngeate distome (BAD) cercariae; **c** Brevifurcate apharyngeate monostome (BAM) cercariae; **d** *Xiphidiocercaria;* **e** Amphistome cercariae; **f** Strigea cercariae; **g** *Longifurcate apharynge*ate distome (LPD) cercariae; **h** Unidentified cercariae

**Fig. 2:**
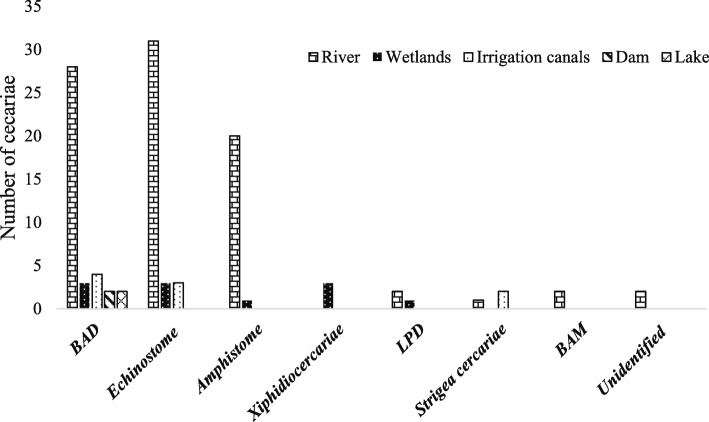
Types and number of trematode infections in different habitat types in the Omo-Gibe River Basin, southwest Ethiopia

A total of eight morphologically distinguishable types of cercariae were recorded from the study sites (Table 2). Trematode infection was recorded in 30(23%) of the sampling sites. Of the 3045 snails examined, 109 (3.6%) released one or more cercariae species. The cercariae species recorded were *Echinostoma*, brevifurcate apharyngeate distome (BAD), amphistoma, brevifurcate apharyngeate monostome (BAM*)*, xiphidiocercaria, longifurcate pharyngeate distome (LPD), strigea cercariae and unidentified cercariae (Plate 1)*.* BAD and *Echinostoma* cercariae were the most abundant cercariae species, accounting 36 and 34% of the total infection, respectively. Most trematode infections were from *B. pfeifferi*, which harboured seven morphologically different cercariae species and accounting for 85% of the total infected snails. *L. natalensis* was infected with echinostoma and xiphidiocercaria and accounted for 10% of the total infected snails. No trematode was recovered form from *B. sudanica.* The highest prevalence of trematode infection was recorded from snails collected from rivers (79%) followed by irrigation canals (10%) (Fig. 2). Snails collected from rivers harboured all cercariae species except xiphidiocercaria*.* On the other hand, snails collected from irrigation canals harbour cercariae of BAD, *Echinostoma* spp.*,* amphistome cercariae, xiphidiocercaria and LPD, whereas Gilgel Gibe 1 Dam and the two shallow lakes had only one type of cercariae.

**Table 2:** Trematode infection in five different snail species collected from Omo-Gibe River Basin, southwest Ethiopia

Snail species	Total number of snails collected	Total number of snails infected	Number of snails infected by cercaria								Infection rate (%)
			BAD	Echinostomes	BAM	Amp	Xip	LPD	Strigea cercariae	Unidentified	
*Biomphalaria pfeifferi*	2010	93	39	26	2	19	_	3	2	2	4.6%
*B.sudanica*	7	_	_	_	_	_	_	_	_	_	_
*Bu.globosus*	148	3	_	3	_	_	_	_	_	_	2.0%
*Bu.forskali*	133	2	_	_	_	2	_	_	_	_	1.5%
*Lymnaea natalensis*	747	11	_	8	_	_	3	_	_	_	1.5%
Total	3045	109	39	37	2	21	3	3	2	2	3.6%

*BAD* Brevifurcate apharyngeate distome, *BAM* Brevifurcate apharyngeate monostome, *Amp* Amphistome, *LPD* Longifurcate pharyngeate distome, *Xip* Xiphidiocercariae

### Environmental factors

The mean values of environmental variables of different habitat types are shown in Table 3. The mean concentration of water conductivity and 5 days biological oxygen demand were higher in irrigation canals and lake sampling points. The highest concentration of conductivity was recorded in Dololo Stream (549 μS/cm) in Jimma Town and a much lower concentration was recorded in Ajikole Stream (42 μS/cm). Similarly, the highest concentration of BOD_5_ was recorded in a Chebera Churchura irrigation canal (84 mg/L), while the lowest concentration of BOD_5_ was recorded in Torbaho Stream (2 mg/L). Human activities such as farming, grazing, open field defecation, bathing and swimming were widely practiced in almost all habitat types, but their intensity varied among different habitats.

**Table 3:** Environmental variables across habitat types. Mean values and range

Variable	River/stream *n =* 100	Wetland *n =* 10	Lake *n =* 10	Dam *n =* 5	Irrigation canal *n =* 5
Water temperature (°C)	23.9 (18.9–33.8)	27.4 (21.6–33.8)	28.4 (25.7–34.7)	26.4 (23.7–31.3)	25.7 (25.5–25.8)
pH	7.2 (5.3–9.1)	7.3 (76.5–9.1)	7.9 (6.8–9.3)	6.6 (5.9–7.1)	7.6 (7.4–7.8)
DO saturation (%)	61.0 (33.0–90.0)	63.0 (33.2–90.0)	98.9 (39.2–376.0)	61.4 (55.3–66.8)	62.0 (60.0–65.0)
EC (μS/cm)	181 (42–549)	159 (74–327)	225 (63–391)	116 (107–131)	231 (229–233)
Turbidity	62.7 (3.7–545.0)	53.5 (14.6–171.0)	227.0 (3.5–66.2)	246.0 (89.0–297.0)	62.6 (53.0–77.0)
PO4^3−^ (mg/L)	0.30 (0.00–2.12)	0.40 (0.01–1.30)	0.12 (0.01–0.53)	0.09 (0.00–0.15)	0.75 (0.57–0.87)
Hardness (mg/L)	40 (12–112)	30 (12–92)	51 (16–86)	20 (16–26)	76 (72–78)
Nitrate	1.70 (0.00–41.00)	0.98 (0.00–3.70)	0.60 (0.00–2.26)	1.50 (0.00–4.70)	1.7 0 (0.96–2.20)
BOD_5_ (mg/L)	13.6 (2.0–65.0)	14.5 (7.0–24.0)	16.0 (18.0–32.0)	11.0 (9.0–14.0)	37.0 (12.0–84.0)
% Pool	57 (0–100)	100 (100–100)	100 (100–100)	100 (100–100)	61 (20–95)
% Riffle	43 (0–100)	0 (0–0)	0 (0–0)	0 (0–0)	39 (5–80)
Grazing	2 (1–3)	3 (2–3)	1 (1–3)	1 (1–2)	2 (1–3)
Farming	2 (1–3)	3 (2–3)	1 (1–2)	2 (1–3)	2 (1–3)
Open Defecation	2 (1–3)	2 (1–3)	2 (1–3)	2 (1–3)	2 (1–3)
Fishing	1 (1–3)	2 (1–3)	1 (1–3)	3 (2–3)	1 (1–1)
Washing	2 (1–3)	3 (2–3)	1 (1–3)	2 (1–3)	2 (1–3)
Bathing	1 (1–3)	2 (2–3)	1 (1–3)	2 (1–3)	1 (1–2)
Swimming	1 (1–3)	2 (1–3)	1 (1–3)	2 (1–3)	1 (1–1)

*pH* logarithmic measure of hydrogen ion concentration, *DO* Dissolved oxygen, *BOD*_*5*_ Five day biochemical oxygen demand, *EC* Electric conductivity, *PO4*^*3−*^ Ortho-phosphate

### Relationship between cercarial infection and environmental factors

**Fig. 3:**
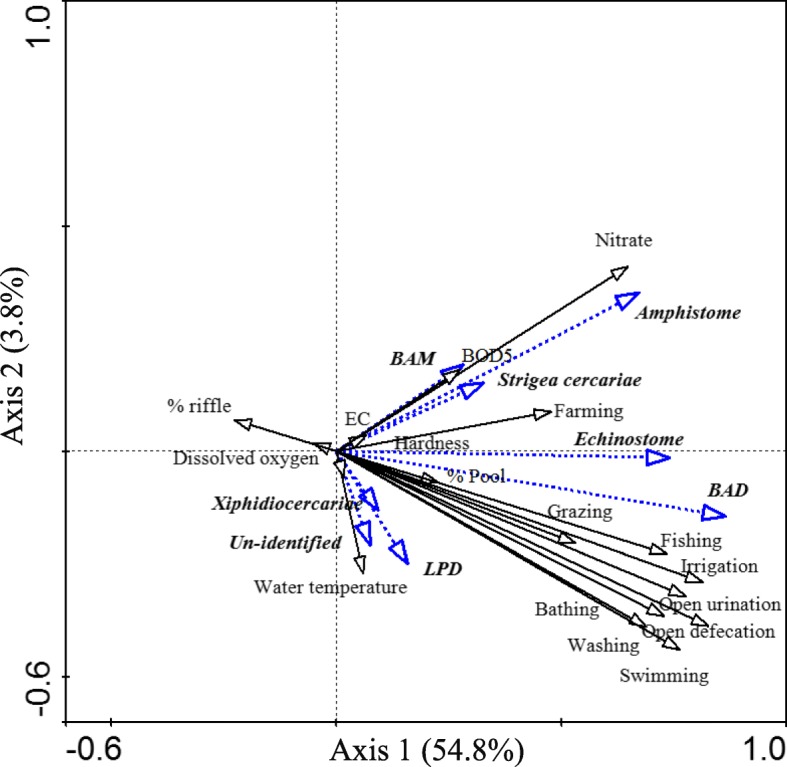
Redundancy analysis (RDA) bi-plot of cercariae and environmental variables in Omo-Gibe River Basin. pH: Logarithmic measure of hydrogen ion concentration; EC: Electric conductivity; BAD: Brevifurcate apharyngeate distome, BAM: Brevifurcate apharyngeate monostome

The first and second canonical axes explained 54.8 and 3.8% of the variation in cercarial infection, respectively. The cercariae-environment correlation of the first two axes was statistically significant in a Monte Carlo permutation test (*P* < 0.05). Based on the RDA, it is evident that human activities were the most important factors determining the abundance of cercariae infection. The first axis was positively correlated with human activities such as open field defecation, open field urination, swimming, livestock grazing, farming and bathing. Moreover, this axis was correlated with nitrate ion concentration. In contrast, riffle flow regime and dissolved oxygen concentration were negatively correlated with the first axis (Fig. 3). As shown in Fig. 1, cercariae were common in cropland and had a limited distribution in shrub land and forests.

## Discussion

The present study documented eight morphologically different cercariae types from five freshwater snail intermediate host species with a 3.6% infection rate that is significantly lower than the 58% infection rate recorded in the study area [8]. The low prevalence of infection in this study could be due to low parasite pressure, making contact between miracidia and snails a rare occurrence [21]. Additional factors may be the difference in snail species observed at different time periods of the year [28] and resistance of some snail species to trematode infection. The snail infection rate may also have been reduced due to the loss of snails during transport from the field sites to the laboratory.

Of the five snail species, *B. pfeifferi* was by far the most infected snail intermediate host species (4.6% infection rate). *B. pfeifferi* was infected by all trematode cercariae except xiphidiocercaria, implying that *B. pfeifferi* had high capability in propagating snail-borne diseases to humans and animals [21]. The brevifurcate apharyngeate distome was the most common type of cercariae in the study area and was recovered only from *B. pfeifferi*. BAD is a mammalian cercaria, whose presence is an indication of *Schistosoma* infection [21, 29]. The variation of infection between *B. pfeifferi* and other snail species could arise from the fact that trematodes are highly specific to their host snails [21]. It could also be due to the abundance of *B. pfeifferi* in the study area, where this species accounted for 66% of the total collection. This is in line with other studies in Ethiopia, where *B. pfeifferi* is the dominant species and principal intermediate host of *Schistosoma mansoni* [30].

In this study, two *B. pfeifferi* snails were infected by both echinostome and amphistome cercariae. This finding is in line with the study done in East Nile locality of Khartoum, Sudan [3], where co-infection in a single snail species was recorded. Overall, snails harbouring co-infections were found less frequently in the study area, indicating that antagonistic interactions may be occurring between different trematodes within the snail, limiting or excluding the establishment of some species [31, 32]. The intensity of co-infecting trematodes is governed by rediae through their antagonistic interspecific interactions against other parasites attempting to infect the same snail host [33, 34]. This is consistent with the fact that interspecific competition for resources and space represents a potentially strong selection pressure for trematodes infecting snail hosts [11]. The low prevalence of brevifurcate apharyngeate distome cercariae in this study might be due to the presence of other trematodes such as echinostome cercariae.

In this study, *L. natalensis* was the second most common species, accounting for 25% of all snails collected and occurring in 30.0% of the surveyed sites. *L. natalensis* was infected by echinostomes and armatae xiphidiocercaria*.* The armatae xiphidiocercariae are intestinal parasites in all groups of vertebrates [9]. *Bu. globosus* was infected by only echinostome cercariae and *Bu. forskalii* by amphistome cercariae. Echinostome cercariae cause echinostomiasis in humans and oral, respiratory and duodenal diseases in livestock. Amphistome cercariae can cause amphistomiasis in humans and domestic animals more commonly in cattle and sheep, causing serious economic loss of the wool, meat and milk industries [34]. Human echinostome infections are most prevalent in Asia, where traditional cultural practices encourage ingestion of raw or undercooked fish, frogs, snakes, or snails and bivalves [10, 35]. Although, consumption of raw/undercooked freshwater molluscs is not common in the study area, human echinostome infections may be acquired through drinking untreated water [36]. Furthermore, human echinostome infection may be expanding to new territories mainly due to new eating habits [37].

Overall, the abundance, occurrence and infectivity of snail species were largely influenced by water quality, sanitation and water contact behaviour of the inhabitants. Water quality variables such as pH, conductivity, BOD_5_ and dissolved oxygen were key determinants of snail occurrence. Our findings are in agreement with studies conducted in southwest Ethiopia [38], which showed that distribution of snail species was associated with high conductivity levels*.* Water bodies polluted by human excreta and sewerage tend to have higher levels of conductivity [39, 40]. *L. natalensis* was encountered in habitats with high BOD_5_ and low dissolved oxygen concentrations. High BOD_5_ and low dissolved oxygen are an indication of organic pollution [40]. Studies have shown that discharge of effluent, pig farming and domestic wastes contributed to the occurrence of freshwater snails in Nigeria [41]. The abundance of trematodes is also positively correlated with habitat condition as most snail species prefer slow moving water for shelter and procreation.

In this study, human activities such as open field defecation, urination, livestock grazing, farming, and swimming were highly correlated with trematode infection. Open field defecation was the main determinant of trematode infections in a mudsnail (*Hydrobia ventrosa*) population inhabiting salt marsh ponds in Iceland [14]. Open defecation is a common practice in sub-Saharan African and Asian countries [42]. In Ethiopia, about 38% of the rural and 9% of the urban population are defecating in fields, forests, bushes, bodies of water, or other open spaces [42] and the characteristically frequent human and livestock contact with freshwater bodies in Ethiopia also results in the release of trematode larvae and disease transmission.

All types of aquatic habitats in the study area yielded one or more types of cercariae. Among these habitats, small rivers and streams had the highest proportion of infected snails (79%), followed by irrigation canals (10%). In the study area, aquatic habitats are commonly used for open defecation and urination, washing clothes, bathing, swimming and washing of farm animals [26]. These practices may result in the release of *Schistosoma* eggs through urine and faeces, where they hatch and release miracidia, which enter into snail hosts and produce cercariae [43]. On the other hand, the rivers, lakes and canals in southwest Ethiopia serve as common watering and grazing grounds for livestock. Hence there is a possibility that cattle, goats and sheep become infected with trematodes. Other sources of contamination may include washing of faeces-contaminated clothes [44], cleaning of the perianal area after defecation during bathing [44, 45], and excrements of wild animal reservoir hosts [44].

Although the researchers were cognizant of the fact that molecular techniques are the most efficient and accurate tools for the identification of snails and associated trematodes, morphological characteristics were used mainly due to resources constraints. The use of morphological characteristics may have failed to identify host-induced phenotypic variations and thus underestimated the diversity of trematode parasites.

## Conclusions

A total of eight morphologically different cercariae types were recovered from five freshwater snail intermediate host species, with a 3.6% infection rate. The abundance, occurrence and infection of snail species were largely influenced by water quality, sanitation and water contact behaviour of the local people. Contaminating human activities such as open field defecation, urination, livestock grazing, farming, and swimming were important predictors of the abundances of cercariae. Therefore, proper containment of excreta (urine and faeces) and reducing human and animal contacts with surface water may significantly reduce cercarial infection and trematode transmission. Nevertheless, additional studies are recommended on the possible role of wild and domestic animals in the transmission of zoonotic trematode infections and the occurrence of echinostomiasis, amphistomiasis, and other less studied trematode infections in humans.

## Supplementary information

**Additional file 1.** Multilingual abstracts in the five official working languages of the United Nations.

## Data Availability

The datasets used during and/or analysed during the current study available from the corresponding author on reasonable request.

## References

[1] Lu XT, Gu QY, Limpanont Y, Song LG, Wu ZD, Okanurak K, Lv ZY (2018). Snail-borne parasitic diseases: an update on global epidemiological distribution, transmission interruption and control methods. Infect Dis Poverty.

[2] Dida OG, Gelder FB, Anyona DN, Matano AS, Abuom PO, Adoka SO (2014). Distribution and abundance of schistosomiasis and fascioliasis host snails along the Mara River in Kenya and Tanzania. Infect Ecol Epidemiol.

[3] Mohammed NAI, Madsen H, Ahmed ARM (2016). Types of trematodes infecting freshwater snails found in irrigation canals in the East Nile locality, Khartoum, Sudan. Infect Dis Poverty.

[4] El-Khayat HM, Ismail NM, Mahmoud KM, Ragb FM, El-Said KM, Mostafa BB (2011). Evaluation of some chemical parameters as potential determinants of freshwater snails with special reference to medically important snails in Egypt. World Acad Sci Eng Technol.

[5] Arshad GM, Maqbool A, Qamar MF, Muhammad S, Bukhari SMH, Hashmi HA, Ashraf M (2011). Prevalence and ecology of freshwater snails in some selected districts of southern Punjab. Pak J Life Soc Sci.

[6] Alemayehu B, Tomass Z (2015). *Schistosoma mansoni* infection prevalence and associated risk factors among schoolchildren in DembaGirara, DamotWoide District of Wolaita zone, southern Ethiopia. Asian Pac J Trop Med.

[7] Farahnak A, Setodeh S, Mobedi IA (2005). Faunistic survey of cercariae isolated from *Melanoides tuberculata* and their role in transmission diseases. Arch Razi Ins.

[8] Mengistu M, Shimelis T, Torben W, Terefe A, Kassa T, Hailu A. Human intestinal schistosomiasis in communities living near three rivers of Jimma town, south western Ethiopia. Ethiop J Health Sci. 2011;21(2):111–18. 10.4314/ejhs.v21i2.69051 PMC3275858 22434990

[9] Tehrani A, Javanbakht J, Khani F, Hassan MA, Khadivar F, Dadashi F, Alimohammadi S, Amani A (2015). Prevalence and pathological study of *Paramphistomum* infection in the small intestine of slaughtered ovine. J Parasit Dis.

[10] Fried B, Thaddeus K, Graczy K, Taman L (2004). Food-borne intestinal trematodiases in humans. Parasitol Res.

[11] Combes C (1982). Trematodes: antagonism between species and sterilizing effects on snails in biological control. Parasitology.

[12] Davis NE (1998). Population dynamics of and larval trematode interactions with *Lymnaea tomentosa* and the potential for biological control of schistosome dermatitis in Bremner Bay, Lake Wanaka, New Zealand. J Helminthol.

[13] Tigga MN, Bauri RK, Deb AR, Kullu SS (2014). Prevalence of snail’s intermediate host infected with different trematodes cercariae in and around Ranchi. Vet World.

[14] Skirnisson K, Glaktionov KV, Kozminsky EV (2004). Factors influencing the distribution of digenetic trematode infections in a mudsnail (*Hydrobia ventrosa*) population inhabiting salt marsh ponds in Iceland. J Parasitol.

[15] Awulachew SB, Yilma AD, Loulseged M, Loiskand W, Ayana M, Alamirew T (2007). Water resources and irrigation development in Ethiopia. (Working Paper 123).

[16] Opisa S, Odiere MR, Jura WG, Karanja DMS, Mwinzi PNM (2011). Malacological survey and geographical distribution of vector snails for schistosomiasis within informal settlements of Kisumu City, western Kenya. Parasit Vectors.

[17] Itagaki H, Suzuki N, Ito Y, Hara T, Wondie T (1975). Study on the Ethiopian freshwater molluscs, especially on identification, distribution and ecology of vector snails of human schistosomiasis. Japan J Trop Med Hyg.

[18] Brown DS (1994). Freshwater snails of Africa and their medical importance.

[19] Harrold NM, Guralnick RP (2010). A field guide to the freshwater mollusks of Colorado.

[20] Ahmed AAM, Ibrahim NA, Idris MA (2006). Laboratory studies on the prevalence and cercarial rhythms of trematodes from Khartoum state. Sultan Qaboos Univ Med J.

[21] Frandsen F, Christensen NO (1984). An introductory guide to the identification of cercariae from African freshwater snails with special reference to cercariae of trematode species of medical and veterinary importance. Acta Trop.

[22] Schell SC (1995). Handbook of trematodes of North America north of Mexico.

[23] Devkota R, Brant SV, Loker ES (2015). The *Schistosoma indicum* species group in Nepal: presence of a new lineage of schistosome and use of the *Indoplanorbis exustus* species complex of snail hosts. Int J Parasitol.

[24] Jayawardena UA, Rajakaruna RS, Amerasinghe PH (2010). Cercariae of trematodes in freshwater snails in three climatic zones in Sri Lanka. Ceylon J Sci Biol Sci.

[25] APHA (1998). Standard methods for the analysis of wastewater.

[26] Mereta ST, Boets P, De Meester L, Goethals PLM (2013). Development of a multimetric index based on benthic macroinvertebrates for the assessment of natural wetlands in Southwest Ethiopia. Ecol Indic.

[27] ter Braak CJF, Šmilauer P. CANOCO Reference Manual and Canoco Draw for Windows User’s Guide: Software for Canonical Community Ordination (Version 4.5). Ithaca; 2002. p. 500. www.canoco.com (Microcomputer Power )

[28] Born-Torrijos A, Poulin R, Raga JA, Holzer AS (2014). Estimating trematode prevalence in snail hosts using a single-step duplex PCR: how badly does cercarial shedding underestimate infection rates?. Parasit Vectors.

[29] Owojori OJ, Asaolu SO, Ofoezie IE (2006). Schistosomiasis: water contact pattern and snail infection rates in Opa reservoir and research farm ponds in Obafemi Awolowo University, Ile-Ife, Nigeria. Int J Zool Res.

[30] Alebie G, Erko B, Aemero M, Petros B (2014). Epidemiological study on *Schistosoma mansoni* infection in Sanja area, Amhara region, Ethiopia. Parasit Vectors.

[31] Sousa WP (1992). Interspecific interactions among larval trematode parasites of freshwater and marine snails. Am Zool.

[32] Keeney DB, Boessenkool S, King TM, Leung TLF, Poulin R (2008). Effects of interspecific competition on asexual proliferation and clonal genetic diversity in larval trematode infections of snails. Parasitology.

[33] MacLeod C, Poulin R, Lagrue C (2018). Save your host, save yourself? Caste- ratio adjustment in a parasite with division of labor and snail host survival following shell damage. Ecol Evol.

[34] Martin GL, Cabrera EC. Morphological characterization of emerging cercariae among *Lymnaeid* snails from Barangay Cawongan, Padre Garcia, Batangas, Philippines. J Parasitol Res. 2018:5241217. 10.1155/2018/5241217. 10.1155/2018/5241217 PMC6151677 30275988

[35] Dodangeh S, Daryani A, Sharif M, Gholami S, Kialashaki E, Moosazadeh M (2019). Sarvi Freshwater snails as the intermediate host of trematodes in Iran: a systematic review. Epidemiol Health.

[36] Xiao X, Dabing L, Tianping W (1995). Studies on mode of human infection with *Echinochasmus liliputanus*. Chinese J Parasitol Parasit Dis.

[37] Toledo R, Esteban JG (2016). An update on human echinostomiasis. Trans R Soc Trop Med Hyg.

[38] Yigezu G, Mandefro B, Mengesha Y, Yewhalaw D, Beyene A, Ahmednur M (2018). Habitat suitability modelling for predicting potential habitats of freshwater snail intermediate hosts in Omo-gibe river basin, Southwest Ethiopia. Ecol Inform.

[39] Mereta ST, Boets P, Bayih AA, Malu A, Ephrem Z, Sisay A (2012). Analysis of environmental factors determining the abundance and diversity of macroinvertebrate taxa in natural wetlands of Southwest Ethiopia. Ecol Inform.

[40] De Troyer N, Mereta ST, Goethals P, Boets P (2016). Water quality assessment of streams and wetlands in a fast growing east African city. Water.

[41] Ayanda OI (2009). Prevalence of snail vectors of schistosomiasis and their infection rates in two localities within Ahmadu Bello University (A.B.U.) campus, Zaria, Kaduna state, Nigeria. J Cell Anim Biol.

[42] Ayalew AM, Mekonnen WT, Abaya SW, Mekonnen ZA (2018). Assessment of diarrhea and its associated factors in under-five children among open defecation and open defecation-free rural settings of Dangla District, Northwest Ethiopia. J Environ Public Health.

[43] Sector WE (2014). Water-based interventions for schistosomiasis control. Pathog Glob Health.

[44] Chala B, Torben W (2008). An epidemiological trend of urogenital schistosomiasis in Ethiopia. Front Public Health.

[45] Grimes JET, Croll D, Harrison WE, Utzinger J, Freeman MC, Templeton MR (2015). The roles of water, sanitation and hygiene in reducing schistosomiasis: a review. Parasit Vectors.

