# Supplementary material for: Environmental determinants of distribution of freshwater snails and trematode infection in the Omo Gibe River Basin, southwest Ethiopia
Source: Infect Dis Poverty. 2019 Nov 20;8:93. doi: 10.1186/s40249-019-0604-y (PMC6865041; doi:10.1186/s40249-019-0604-y)
Supplement: Supplementary file 1 — Additional file 1. Multilingual abstracts in the five official working languages of the United Nations. [file 40249_2019_604_MOESM1_ESM.pdf]

Translation of the abstract into the five official working languages of the United Nations

المحددات البيئية لانتشار حلزون المياه العذبة وعدوى المثقوبة في حوض نهر أومو جيبي (Omo Gibe)، جنوب غرب إثيوبيا

سيد تيكو ميريتا، جمال بيدوي، ديلناسو يوهالو، بلايهون مانديفرو، ييهون عبدي، ديشاسا تيجن، وندوسن بيرك، وركو لاجيز مولات، هيلموت كلوس

ملخص
المعلومات الأساسية: يعد تحديد معدلات العدوى لتجمعات الحلزونات أحد الأدوات الأساسية لدراسات الأوبئة الخاصة بالأمراض المنقولة عن طريق الحلزون. اخترنا، في هذه الدراسة، تحديد عدوى المثقوبة لحلزون المياه العذبة في حوض نهر أومو جيبي (Gibe-Omo)، جنوب غرب إثيوبيا.
الطرق: جمعنا عينات لحلزونات من 130 موقع رصد في البحيرات والمستنقعات والأنهار والخزانات وقنوات الريالتي تم مسحها خلال موسم الجفاف (من مارس إلى مايو) في 2016. خضعت عينات الحلزون لفحص عدوى المثقوبة عن طريق عزل الذانبة مباشرة بعد التجميع. تم تقييم الظروف البيئية ونوعية المياه وممارسات التعرض لمياه الشرب وغيرها من الأنشطة البشرية في كل موقع مسح. تم استخدام تحليل تكرار (RDA) لدراسة العلاقة بين الإصابة بعدوى الذانبة والمتغيرات البيئية. تم اختبار الدلالة الإحصائية للقيم الخاصة وعلاقتها ببيئة الذانبة الناتجة عن تحليل التكرار باستخدام التحولات في طريقة مونتي كارلو عند 499 من التحولات.
النتائج : تم جمع إجمالي 3107 من الحلزونات التي تنتمي إلى خمس فصائل. أكثر الفصائل وفرة كانت حلزون iomphalaria pfeifferiB، ممثلة إجمالي 66٪ من المجموع الكلي. تم العثور على مجموع 109 (3.6٪) من الحلزونات مصابة بعدوى المثقوبة (الذانبة). وُجد أن أعلى الفصائل إصابة هي Biomphalaria pfeifferiفهي تحمل 85٪ من جميع عدوى المثقوبة. تم تسجيل ما مجموعه ثمانية أنواع مختلفة من الناحية الشكلية من الذانبة، والتي شملت: Echinostoma، *brevifurcate* pharyngeate diastome، amphistome cercariae، *brevifurcate* monostome apharyngeate، *xiphidiocercariae*، *pharynge longifurcate* ate*distome*، *strigea* cercariae وذانبة غير محدَدة.تعد*brevifurc*ate apharyngeate diastome، و Echinostoma أنواع الذانبة الأكثر وفرة، وهو ما يمثل 36.8 ٪ و34.0 ٪ من جميع أنواع العدوى على التوالي. كان متوسط الـتركيز لتوصيل المياه والطلب البيولوجي للأكسجين لمدة خمسة أيام أعلى في قنوات الري ونقاط أخذ العينات بالبحيرة. كانت الأنشطة البشرية مثل التغوط في العراء والتبول ورعي الماشية والزراعة والسباحة مرتبطة ارتباطاً وثيقاً بعدوى المثقوبة.
الاستنتاجات: تأثرت معدلات وفرة وحدوث وعدوى أنواع الحلزونات إلى حد كبير بالنوعية الطبيعية والكيميائية للمياه والمرافق الصحية وسلوك تعامل السكان مع الماء. مثَّلت الأنشطة البشرية مثل التغوط في العراء والتبول ورعي الماشية والزراعة والسباحة مؤشرات هامة بوجود الذانبة. لذلك، يجب نشر الوعي من أجل الاحتواء الصحيح للفضلات (البول والبراز) وتقليل التعامل البشري والحيواني بالمياه السطحية لتقليل انتقال الأمراض المنقولة عن طريق الحلزون.

Translated from English version into Arabic by Hager Ryan, revised by Amal Imam, through

TRANSLATORS
WITHOUT BORDERS

埃塞俄比亚西南部Omo Gibe河流域淡水螺分布和吸虫感染的环境决定因素

Seid Tiku Mereta，Jemal Bedewi，Delenasaw Yewhalaw，Belayhun Mandefro，Yihun Abdie，Dechassa Tegegne，Wondwosen Birke，Worku Legesse Mulat，Helmut Kloos

摘要
引言：确定螺的感染率是螺传疾病流行病学研究的基本工具之一。本研究中，我们确定了埃塞俄比亚西南部Omo-Gibe河流域淡水螺的吸虫感染情况。
方法：我们收集了2016年干旱季节（3～5月）湖泊、湿地、河流、水库和灌溉渠的130个观测点的淡水螺样本。随后采用逸蚴法检查螺的吸虫感染情况。在每个调查点检查居住环境、水质、人类接触水的方式以及其他人类活动。采用冗余分析（RDA）评价尾蚴感染和环境变量之间的关系。使用499个排列的蒙特卡罗置换检验(Monte Carlo permutation)对RDA生成的特征值和尾蚴-环境的相关性进行显著性分析。

结果：共收集隶属于5个种的3107只螺，菲氏双脐螺（66%）最多。其中109只（3.6%）螺感染吸虫（尾蚴）。菲氏双脐螺的感染最严重，占感染螺的85%。总共记录了8种形态不同的尾蚴，包括棘口吸虫属尾蚴、短尾叉具咽双盘尾蚴（brevifurcate apharyngeate distome，BAD）、对盘尾蚴、短尾叉具咽单盘尾蚴（brevifurcate apharyngeate monostome）、剑尾蚴、长尾叉无咽双盘尾蚴（longifurcate pharyngeate distome）、鸮形吸虫属尾蚴和未分类尾蚴。其中最多的是BAD和棘口吸虫属尾蚴，分别占所有感染螺的36%和34%。灌溉渠和湖泊采样点的平均水电导率和五日生化需氧量较高。露天排便、排尿、放牧、耕种和游泳等人类活动与吸虫感染高度相关。
结论：螺种的丰度、发生率和感染率在很大程度上受居民水理化质量、卫生条件和接触水行为的影响。人类活动，例如露天排便和排尿、放牧、耕种和游泳是尾蚴丰度的重要预测因素。因此，应适当控制排泄物（尿液和粪便），并减少人和动物与地表水的接触，以减少螺传播的疾病。

Translated from English version into Chinese by Cong-Shan Liu, edited by Pin Yang

Les déterminants environnementaux de la propagation des gastéropodes d'eau douce et de la trématodose dans la rivière Omo-Gibe en Éthiopie

Seid Tiku Mereta, Jemal Bedewi, Delenasaw Yewhalaw, Belayhun Mandefro, Yihun Abdie, Dechassa Tegegne, Wondwosen Birke,Worku Legesse Mulat, Helmut Kloos

Résumé
Contexte: L'établissement du taux d'infection des populations de gastéropodes est l'un des instruments de base dans les études épidémiologiques sur les maladies transmises par ces derniers. Dans cette étude, nous avons décidé d'identifier la trématodose chez les gastéropodes d'eau douce dans la rivière Ome-Gibe en Éthiopie.
Méthodes: Nous avons collecté des échantillons de gastéropodes sur 130 sites d'observation (lacs, milieux humides, rivières, réservoirs et canaux d'irrigation) étudiés pendant la saison sèche (du mois de mars au mois de mai) de 2016. Les échantillons de gastéropodes ont été analysés pour la trématodose en éliminant la cercaire immédiatement après le prélèvement. Nous avons analysé les conditions d'habitat, la qualité de l'eau, les modes de contact de l'Homme avec l'eau et toutes les autres activités humaines sur chaque site d'étude. Nous avons conduit une analyse canonique de redondance (ACR) afin d'étudier la relation entre l'infection cercarienne et les variables environnementales. La pertinence statistique de la valeur propre et des corrélations entre l'environnement et la cercaire générées par l'ACR ont été analysées à l'aide des permutations Monte Carlo avec 499 permutations.
Résultats: Au total, 3107 gastéropodes appartenant à cinq espèces différentes ont été prélevés. Le *Biomphalaria pfeifferi*était l'espèce la plus présente, représentant 66% du nombre total de prélèvement. Au total, 109 (3,6%) des gastéropodes étaient atteints d'une trématodose (cercaire). Nous avons découvert que les *Biomphalaria pfeifferi* étaient les plus infectés, représentant 85% de toutes les trématodoses. Nous avons répertorié un total de huit espèces de cercaires différentes morphologiquement : *l'Echinostoma,* le distome pharyngé brevifurcate , l'amphistome cercariae, le monostome apharyngé brevifurcate , le xiphidiocercariae, *le distome*pharyngé longifurcate, le strigea cercariae et une cercaire non-identifiée. Brevifurcate apharyngeate diastome et *Echinostoma* cercariae étaient les cercaires les plus présentes, représentant respectivement 36,8% et 34,0% de toutes les infections. La concentration moyenne de conductivité de l'eau et la demande biologique en oxygène sur 5 jours étaient supérieurs dans les canaux d'irrigation et les points de prélèvement lacustres. Les activités humaines telles que la défécation et la miction en plein air, le pâturage du bétail, l'agriculture et la natation sont en étroite corrélation avec la trématodose.
Conclusions: L'abondance, l'occurrence et le taux d'infectiondes espèces de gastéropodes sont largement influencés par la qualité physicochimique de l'eau, l'assainissement et les modes de contact de la population avec l'eau. Les activités

humaines, telles que la défécation et la miction en plein air, la pâturage du bétail, l'agriculture et la natation représentaient des indicateurs importants de l'abondance de cercaire. Par conséquent, il est nécessaire de sensibiliser au confinement approprié des excréments (urine et fèces) et à la réduction des contacts des animaux et des êtres humains avec la surface de l'eau afin de réduire la transmission de maladies dues aux gastéropodes.

Translated from English version into French by Florie Ulian, revised by Marie Lamardelle, through

TRANSLATORS
WITHOUT BORDERS

Детерминанты окружающей среды, отвечающие за распределение пресноводных улиток и трематодоза в бассейне рек Омо-Гибе на юго-западе Эфиопии

Сеид Тику Мерета, Джемал Бедеви, Деленасав Евхалав, Бэлаихун Мандефро, Йихун Абдие, Дечасса Тегегне, Вондвосэн Бирке, Ворку Легессе Мулат, Хельмут Клоос

Реферат

Предпосылки: Определение уровня заражения популяций улиток является одним из главных инструментов эпидемиологических исследований заболеваний, распространяемых улитками Целью данного исследования является выявление трематодоза у пресноводных улиток в бассейне рек Омо-Гибе на юго-западе Эфиопии.

Методы: Мы собрали образцы улиток из 130 мест наблюдения в районе озёр, болот, рек, водохранилищ и оросительных каналов при проведении исследования в период засухи с марта по май 2016 года. Непосредственно после сбора образцы улиток проверялись на заражение трематодозом по церкариальным выделениям. В каждом месте наблюдения были оценены условия обитания, качество воды, возможности контакта человека с водой и другие виды человеческой деятельности . Для определения связи между церкариальной инфекцией и переменными окружающей среды использовался анализ избыточности. Статистическая значимость собственных значений и корреляции среды церкарий, создаваемые при анализе избыточности, тестировались с помощью пермутаций Монте-Карло на 499 пермутациях.

Результаты: В общей сложности было собрано 3107 улиток пяти видов. Наиболее многочисленно был представлен вид *Биомфалярия Пфейффера*, составивший 66% от всех собранных улиток. В целом, 109 (3,6%) улиток оказались зараженными трематодозом (церкариальным). *Биомфалярия Пфейффера* была определена как наиболее заразная, она отвечает за развитие 85% всех трематодозных инфекций. Всего было зарегистрировано 8 разных морфологических типов церкарии, включая*эхиностому,* бревифуркат фарингеатная диастома, амфистомная церкария, бревифуркат моностом афарингеата, ксифидиоцеркария, *продольный фарингец*ate дистома, стригея церкария и неопознанная церкария. Бревифуркат ная диастома и *эхиностомная* церкарии были представлены наиболее многочисленно, на них пришлись, соответственно, 36.8% и 34.0% от всех выявленных случаев заражения. Средняя концентрация проводимости воды и пятидневная потребность биологического кислорода были выше в оросительных каналах и в местах отбора из озер. Человеческая деятельность, такая как, например, дефекация на открытой местности, уринация, выпас скота, фермерство и плавание очень тесно связаны с трематодозной инфекцией. .

Выводы: Численность, распространённость и уровень инфицирования различных видов улиток в большой степени зависят от физико-химических свойств воды, санитарных условий и контакта обитателей с водой. Человеческая деятельность, такая как дефекация и уринация на открытой местности, выпас скота, ведение

сельского хозяйства и плавание обуславливали высокую численность церкарий. Поэтому необходимо повысить осведомленность о надлежащем сборе экскрементов (мочи и кала), а также о сокращении человеческих и животных контактов с поверхностью воды, чтобы препятствовать распространению заболеваний, переносимых улитками.

Translated from English version into Russian by Maria Petrenko, revised by Alexander Somin, through

TRANSLATORS
WITHOUT BORDERS

Determinantes ambientales en la distribución de caracoles de agua dulce e infecciones por trematodos en la cuenca del río Omo-Gibe, al suroeste de Etiopía

Seid Tiku Mereta, Jemal Bedewi, Delenasaw Yewhalaw, Belayhun Mandefro, Yihun Abdie, Dechassa Tegegne, Wondwosen Birke,Worku Legesse Mulat, Helmut Kloos

Resumen
Introducción: La determinación de los índices de infección en las poblaciones de caracoles es una de las herramientas básicas para los estudios epidemiológicos de las enfermedades transmitidas por los caracoles. En el presente estudio, optamos por determinar la infección por trematodos en los caracoles de agua dulce de la cuenca del río Omo-Gibe al suroeste de Etiopía.
Métodos: Recolectamos muestras de caracoles en 130 sitios de observación: en lagos, humedales, ríos, embalses y canales de irrigación, los cuales se inspeccionaron durante la estación seca (de marzo a mayo) en el año 2016. Las muestras de caracoles se examinaron por infecciones de trematodos, mediante descamación cercarial, inmediatamente después de la recolección. Se evaluaron las condiciones de hábitat, la calidad del agua, las prácticas de contacto humano con el agua y otras actividades humanas en cada sitio de la encuesta. Se usó un análisis de redundancia (RDA), para examinar la relación entre la infección cercarial y las variables ambientales. La significación estadística de los valores propios y las correlaciones cercaria-ambiente generadas por la RDA se probaron utilizando permutaciones Monte Carlo a 499 permutaciones.
Resultados: Fueron recolectados un total de 3107 caracoles pertenecientes a cinco especies distintas. La especie más abundante fue *Biomphalaria pfeifferi*, representando el 66% en el total de la recolección. En total, 109 (3,6%) de los caracoles se encontraban infectados con trematodos (cercariae). Se encontró que la bacteria *Biomphalaria pfeifferi*resultó ser la más infectada, representando el 85% de todas las infecciones por trematodos. Se registraron un total de ocho tipos de cercariae morfológicamente diferentes, que incluían: *Echinostoma,* brevifurcate pharyngeate diastome, amphistome cercariae, brevifurcate apharyngeate monostome, xiphidiocercariae, *longifurcate pharynge*ate distome, strigea cercariae ycercariae sin identificar. Brevifurcate apharyngeate diastome, y*Echinostoma* cercariae fueron las cercariae más abundantes, representando el 36,8% y el 34,0% de todas las infecciones, respectivamente. La concentración media de conductividad del agua y la demanda biológica de oxígeno durante cinco días fueron mayores en los canales de riego y en los puntos de muestreo de los lagos. Las actividades humanas, como la defecación en campo abierto, la micción, el pastoreo de ganado, la agricultura y la natación, estaban altamente correlacionadas con la infección por trematodos.
Conclusiones: La abundancia, la ocurrencia y las tasas de infecciónde las especies de caracoles fueron influenciadas en gran medida por la calidad fisicoquímica del agua, el saneamiento y el comportamiento de contacto de los habitantes con el agua. Actividades humanas como la defecación y la micción en campo abierto el pastoreo de ganado, la agricultura y la

natación eran importantes vaticinadores de la abundancia de cercariae. Por lo tanto, una creación de conciencia debe implementarse para la contención adecuada de las excretas (orina y heces) y reducir los contactos humanos y animales con las aguas superficiales para reducir la transmisión de enfermedades transmitidas por caracoles.

Translated from English version into Spanish by Maria Luz Puerta, revised by Daniel Casado Rodriguezi, through

TRANSLATORS
WITHOUT BORDERS
